# Working hours, social engagement, and depressive symptoms: an extended work-life balance for older adults

**DOI:** 10.1186/s12889-023-17072-x

**Published:** 2023-12-06

**Authors:** Young-Mee Kim, Soong-nang Jang, Sung-il Cho

**Affiliations:** 1 Department of Public Health Science, Graduate School of Public Health, Institute of Health and Environment, Seoul National University, 1 Gwanak-ro, Gwanak‐gu, Seoul, 08826 South Korea; 2 Red Cross College of Nursing Chung-Ang University, Seoul, South Korea

**Keywords:** Work-family conflict, Work‐life balance, Depression, Mental health, Resilience, Healthy aging

## Abstract

**Background:**

In recent years, researchers have been examining the impact of work-life balance (WLB) on mental health, considering it as a potential risk factor. However, it remains unclear whether the traditional understanding of WLB applies to older adults who worked for fewer hours before full retirement and whose children are likely to be independent adults. Therefore, this study aims to propose a modified form of WLB specifically for older adults. Within this context, we hypothesize that an optimum balance between working hours and social engagement protects against depressive symptoms among older adults.

**Method:**

We conducted an analysis using data on 5,751 Korean adults older than 55 years from the Korean Longitudinal Study of Aging 2016. Multivariate logistic regression analysis was used to evaluate the relationships among working hours, social engagement, and depressive symptoms.

**Results:**

Older adults who worked fewer than 35 h per week were less likely to experience depressive symptoms than were non-working older adults and those working 35 h or more per week. Additionally, older adults with a high level of informal social participation, thus occurring almost every day or two to three times per week, were less likely to experience depressive symptoms than were those with a low level of such participation (once a month or less). Furthermore, depressive symptoms were less frequent among those who worked fewer than 35 h per week and engaged in a high level of informal social participation compared to non-working older individuals and those with a low level of informal social participation.

**Conclusions:**

Maintaining an optimal number of working hours and degree of social engagement are necessary to minimize the risk of depressive symptoms in older adults. Based on these findings, we suggest that fulfillment for work and life and their balance are important for older adults and propose work–life fulfillment balance.

## Introduction

Worldwide, increasing numbers of individuals aged 55 and older remain in the labor force [1]. This highlights the evolving nature of the international workforce. The increased participation of older adults in the labor force is attributable to increased life expectancy, more years of good health, delayed life transitions, less physically demanding work, and higher educational attainment [2]. Of the Organization for Economic Co-operation and Development countries, Korea has the second-highest labor-force-participation rate for adults aged ≥ 65 years (37.3%) [1] and the highest effective age of labor-market exit for both women and men (72.3 years) [3]. The large time gap of 11.3 years between the average retirement age (61 years) and the effective age of labor-market exit in Korea suggests that individuals are reemployed in daily or temporary jobs during that time, or are self-employed [4].

While the Korean employment rate for older adults is relatively high, the poverty rate for individuals aged 65 and over significantly exceeds the rates found in other OECD countries, standing at 43.4% compared to the OECD average of 13.1% [5]. The underlying vulnerabilities within Korea’s public pension system serve as the foundation for this issue [6]. Uniquely in Korea, the principal income streams for older adults aged 65 and above are not national pensions, rather labor earnings and family allowances [7]. Although industry has grown, job opportunities for older adults have not grown at the same rate. Thus, many such adults perform temporary, occasional, and manual tasks, which typically offer limited compensation [8].

Although economic necessity thus forces many older Koreans to work, this is not the only motivator. A 2022 survey by the Korean Statistical Office revealed that 53.3% of such adults work primarily for financial reasons, whereas 37.3% find fulfilment and joy in their work [9]. An additional 5.2% work to stave off idleness. Remarkably, this indicates that approximately 42.5% of such adults are motivated by reasons other than money. Individuals work not only to earn money but also to satisfy their psychological and social needs [10]. Employment promotes healthy aging; providing continued employment is a public-health priority [11]. The activity theory of aging suggests that sufficient physical activity and social interaction are essential for healthy aging [12]. Previous studies found that older adults who continued to work after retirement were healthier than those who retired [13]. Occupational and work-related activities are associated with increased physical activity [14]. Additionally, prolonging the working life may increase individuals’ social participation and provide social support, leading to improved health of older adults [11].

Previous studies revealed significant associations between work-related factors (other than employment) and health. Job stress and long working hours are risk factors for chronic diseases [15, 16]. Several recent studies have found that work-life imbalance is a risk factor for chronic diseases [17]. Work-life balance (WLB) refers to a state of equilibrium between the demands of a person’s job and personal life [18]. WLB is often compared to a similar term, thus work-family balance, but the former includes community, social, religious, and leisure activities [19]. Work-life imbalance negatively affects workplace productivity and worker health. Previous studies showed that a poor WLB was associated with depressive symptoms and musculoskeletal disorders [20, 21].

Various theories regarding WLB have emerged since the 1970s. Beginning with the Intuitive Segmentation theory, the scholarly literature broadened to include the Spillover, Border, Boundary, and Enrichment theories [19]. Among the multitude of theories, Boundary and Border theories stand out as the primary foundational frameworks frequently employed in various studies to explore different aspects of WLB [22]. According to these theories, work and non-work are distinct life domains demarcated by cognitive, physical, and behavioral boundaries [23]. However, the work and non-work domains interact as workers transition the boundaries between work and home. In light of this, both theories explore how individuals establish, maintain, negotiate, and traverse the boundaries between their professional and familial roles [24]. Building on this, the Border Theory defines WLB as satisfaction and optimal functioning in both professional and domestic settings, with minimal role conflict [25].

We prefer the Enrichment theories that, especially from the viewpoint of older adults, emphasize the potential synergistic relationship between work and life rather than the traditional conflict-centric perspective of WLB. Powell & Greenhaus (2006) developed the Enrichment Theory to elucidate how enriching experiences link work to family and family to work [26, 27]. Enrichment is defined as the process by which experiences in one role enhance the quality of life in another role. These theories underscore the growth and maintenance of resources as viewed through the lens of resource conservation [28, 29]. A previous study suggested that work-life enrichment was attributable to resource generation via proficient work execution, in turn heightening satisfaction and promoting positive outcomes in personal life [30]. Within this context, the key resources include positive emotions, psychological resilience, skills, adaptability, and social support. Furthermore, the concept of work-life enrichment implies that active engagement in both professional and familial roles provides a cushion that mitigates potential distress in either domain [15].

Existing research on WLB theory and the health impacts thereof has focused predominantly on younger workers, who are often juggling job commitments and childcare. The experiences of older workers, many of whom may be nearing retirement or have already retired and consequently have fewer obligations, have received less attention. This ignores the unique challenges and perspectives of older individuals. However, some relevant studies have appeared; one notable example is the cohort study of Cho and Chen who found a reciprocal correlation between the illnesses and disabilities of workers aged 55 and over and work–family conflict (WFC) [31]. A bidirectional relationship was apparent: For older workers, WFC escalated health problems and imposed functional limitations. Conversely, those with more health problems were at greater risk of more WFC. Also, some studies have addressed WLB differences across various age groups. Younger individuals tend to experience more frequent WLB disruptions compared to older subjects [32, 33]. Of all workers, those aged between 55 and 64 and those aged 65 and older consistently reported the highest levels of WLB satisfaction [34].

Both work- and life-related factors impact the health of older adults. While employment has positive effects on the health, cognition, and physical function of older adults [35], unemployed individuals have a higher risk of depression compared to precarious workers [36]. Additionally, changes in employment status, including retirement, affect the health outcomes of older adults [4]. Several studies have shown retirement to be an influencer, rather than a result, of poor health [37]. However, studies have reported contradictory findings regarding the effects of retirement on health, with some reporting beneficial effects of retirement on psychological well-being [38]. Second, studies have found that longer working hours were associated with negative health outcomes in older individuals [39]. The relationships between working hours and poor physical health were less marked in older workers than in younger workers [40]. Finally, older adults with active social engagement, including in leisure activities, had better health compared to those lacking social engagement [41].

Previous studies of work- and life-related factors had several limitations. First, there are limited number of studies exploring the interplay between work and life and the impact thereof on the health of older adults. Second, although most previous studies of work- and life-related factors classified individuals as employed or unemployed, or as having or lacking social engagement, they did not explore the number of working hours or degree of social engagement required to mitigate the health risks. Third, previous studies did not include people from the general older population, and included workers and retirees but excluded non-workers [39]. A significant proportion of older adults are non-workers, who should be included in studies of WLB.

Depression and depressive symptoms are prevalent among older populations [42], with roughly 2% suffering from major depression and 10–15% experiencing significant depressive symptoms [43]. Older adults with depression exhibit loss of interest, somatic symptoms, and cognitive changes; furthermore, they have a higher risk of completed suicide compared to younger adults [44]. Several factors, including age, health conditions, and socioeconomic status, influence depressive symptoms in older people [45]. Although a poor WLB is a well-known risk factor for depression among younger adults [20], this possibility has not been explored as a potential risk factor for depression among older adults. Furthermore, the relationships among work, social engagement, and depression in older adults have not been established. As discussed previously, this may be because the current concept of WLB is not directly applicable to older adults. Additionally, data pertaining to the non-standard employment of older adults (short-term, part-term, zero-hour contracts) and the non-working population are not available. However, a holistic understanding of the relationships among work, social engagement, and depression in older adults is essential to develop strategic policies and interventions promoting healthy aging.

In this study, we propose a modified WLB concept for older adults that considers the aging process and parameters that reflect working hours and social engagement. Specifically, from an Enrichment perspective, we propose that work- and life-related “fulfillment”, and the balance thereof, are crucial for older adults. We hypothesized that increased participation of the older population in occupational and social activities may prevent depressive symptoms. We further hypothesized that desirable working hours and sufficient social engagement can protect against depressive symptoms. Our aims were to determine whether working hours are significantly related to depressive symptoms, whether depressive symptoms are more common among older adults with little social engagement, and whether an optimum balance between working hours and social engagement protects against depressive symptoms among older adults.

## Methods

### Sample

We used nationally representative cross-sectional data from the sixth wave (2016) of the Korean Longitudinal Study of Aging (KLoSA) conducted by the Korean Labor Institute and Korea Employment Institute Information Service. KLoSA was performed in 2006, with follow-up surveys performed in every even-numbered year thereafter to provide data for developing socioeconomic policies related to population aging [46]. The study collects sociodemographic and family-related data, as well as data on employment status, working conditions, social engagement, health behaviors, and health status. Multistage stratified probability sampling based on geographical area was used. The sixth survey followed up 6,618 participants (64.5% of the original cohort) using computer-assisted personal interviewing. We analyzed data from adults aged ≥ 55 years without limitations in activities of daily living (ADL). We excluded 550 individuals from the analysis due to insufficient income or depressive symptom data. Therefore, data from 5,751 adults (86.9% of the total sample of 6,618 individuals), including 2,492 men and 3,259 women, were analyzed. This study received ethical approval from the Institutional Review Board (IRB) of the Public Institutional Bioethics Committee designated by the Ministry of Health and Welfare (Approval No. P01-201911‐22‐002), and was conducted in accordance with the Declaration of Helsinki. Informed consent was obtained from all participants by trained interviewers.

### Study variables

#### Working hours

Self-reported working hours were recorded based on the responses to the following question: “On average, how many hours do you work per week, including overtime but excluding mealtimes?” Weekly working hours were categorized as 0 (non-working), 1–34, 35 − 54, or ≥ 55 h based on the European Foundation for the Improvement of Living and Working Conditions definition of part-time work (< 35 h per week) and previous studies (≥ 55 h per week) of overtime [47, 48]. Work exceeding 55 h is designated as overtime following Kivimäki (2015), who found that more than 55 working hours per week was associated with increased risks of stroke and incident coronary heart disease [48].

### Social engagement

Social engagement is defined as the performance of meaningful social roles for either leisure or productive activity [49]. Many scholars use the term to refer to participation in both formal and informal social groups; this is then synonymous with social participation [50, 51]. Researchers commonly evaluate participation using two distinct dimensions, of which the formal dimension includes attending meetings, groups, or clubs; religious participation; and volunteering [51, 52]. The informal dimension encompasses social interactions with friends, neighbors, and relatives. Some researchers have delved deeper. For example, Brand (2008) highlighted six potential spheres of participation: church-affiliated groups; charitable organizations; youth groups; civic, business, political, or neighborhood organizations; professional groups; and engagement in leisure activities [53]. Thus, evaluation of social engagement typically requires identification of specific activities and quantitation of the extents of involvement [51].

We adopted a similar methodology; we divided social engagement into formal and informal participation, evaluated using the following questions. Formal participation was evaluated by the following question: “Do you participate in (1) religious meetings; (2) social gatherings with friends or neighbors; (3) leisure, cultural, or sports groups; (4) hometown or family councils; (5) volunteer groups; (6) political parties, non-government organizations, or interest groups; (7) or other organizations?”. Based on the responses to each item (*yes* or *no*), formal participation was recorded as low, medium, or high. Low level corresponds to no involvement; medium level corresponds to involvement in one activity; and high level corresponds to involvement in two or more activities. Informal participation was assessed using the following questions: “Do you have close friends, relatives, or neighborhood friends?” and “How often do you get together with them?”. The responses to these questions were scored from 1 to 10 (1 = *almost every day*, 10 = *no contact or meetings*). Informal participation was classified as low, medium, or high. Individuals who responded *almost every day* or *two to three times a week* were considered to have a high level of informal participation; those who responded *once a week* or *twice a month* were considered to have a medium level of informal participation; and those who responded *once a month*, *once every 2 months*, *once or twice a year*, *three to five times a year*, *I hardly ever see them*, or *there is no such person* were considered to have a low level of informal participation.

### Depressive symptoms

The presence of depressive symptoms was evaluated using the Center for Epidemiologic Studies Depression 10-item scale (CES-D10), which is widely used to screen for depressive symptoms [54, 55]. Of the 10 items, 8 are phrased negatively (e.g., loss of interest and feeling depressed), and two positively (e.g., generally satisfied and feel pretty good). Each item is scored from 0 (*occasionally or < 1 per day in the past week*) to 3 (*almost always or 5–7 days*); reverse scoring is used for positively phrased items. Items scored as ≥ 1 (*sometimes or on 1–2 days*) were coded as 1. Older adults with positive responses to four or more items are considered to have depressive symptoms [55].

### Covariates

The sociodemographic covariates included age (55–59, 60–69, 70–79, or ≥ 80 years), sex (male or female), marital status (separated, divorced, or widowed/ living with a spouse), educational status (primary or less, middle school, high school, or college or higher), income quartile (first quartile = poorest), household assets (first quartile = poorest), residential area (metropolitan, urban, or rural), and religious (yes or no). Family ties included number of children who they meet weekly (none or ≥ 1) and number of children who phone or mail weekly (none or ≥ 1). Work-related factors, such as and job-related demands and physical requirements of the job, were included as covariates because of their reported associations with depressive symptoms [56]. Job-related demands and physical requirements of the job were evaluated based on the responses to the statements “My job requires me to perform increasingly difficult tasks” and “My job requires lots of physical effort”, respectively. Individuals who responded to either of the aforementioned statements with *always or most of the time* were considered to have high job-related demands. Individuals who provided other responses (*some of the time* or *none or almost none of the time*) were considered to have low job-related demands. The presence of family caregiving was assessed by asking respondents whether they had any co-residing or non-co-residing family members who provided assistance with Activities of Daily Living (ADL).

We also recorded health-related variables related to depressive symptoms, including self-rated health (SRH), limitations in instrumental ADLs (IADL), work-life limitations due to health problems, cognitive problems, number of chronic diseases, body mass index (BMI), smoking, alcohol consumption, and exercise. SRH was evaluated using the following question: “In general, would you say your health is excellent, very good, good, fair, or poor?”. Based on the answers, SRH was classified as good (*excellent* or *good*) or poor/average (*poor*, *very poor*, or *fair*). Work-life limitations due to health problems were evaluated by asking whether or not the participants faced difficulty in working or social engagement because of their health problems. Limitations in instrumental ADL was evaluated using a 10-item Korean IADL scale [57]. The scale includes related to going out for short walks, personal grooming, handling transportation, managing finances, doing housework, preparing meals, using the telephone, shopping, doing laundry, and managing medications. Individuals who were partly or completely dependent for a given activity were considered to have IADL limitations. Cognitive function was evaluated using the Korean version of the Mini-Mental State Examination, which has been validated in previous studies [58]. The maximum score is 30 points; individuals with ≥ 25 or < 25 points are classified as having normal cognition and possible dementia or dementia, respectively. Participants could report the presence of one or more of eight physician-diagnosed diseases: hypertension, diabetes, lung diseases, cardiac disorders, cerebrovascular disease, psychiatric diseases, liver diseases, and arthritis. The responses were categorized as no, 1, or ≥ 2 diseases. Based on the BMI, participants were categorized as normal or low weight (< 23 kg/m^2^), overweight (23–24.9 kg/m^2^), or obese (≥ 25 kg/m^2^), in accordance with the World Health Organization classification of BMI for Asian populations [59]. Smoking status was recorded as non-smoker (including former smoker) or current smoker. Alcohol consumption and regular exercise were reported as *yes* or *no*.

### Statistical methods

The chi-square test was performed to compare work- and life-related factors among the different age groups. Multivariate logistic regression analysis was performed to evaluate the relationships among working hours, social engagement, and depressive symptoms, after controlling for covariates such as age, sex, marital status, educational level, income, assets, residential area, family ties, caregiving, job-related demands, physical requirements of the job, SRH, work-life limitations due to health problems, IADL limitations, cognitive function, number of chronic diseases, and health behaviors. Multivariate logistic regression analysis was also conducted to evaluate sex differences in the effects of working hours and social engagement on depressive symptoms. Finally, we constructed a composite variable to capture WLB by integrating the numbers of working hours with the extent of social engagement, with a focus on informal participation. We then performed logistic regression. Various combinations of the number of working hours (0, < 35, 35–54, and ≥ 55 h) and degree of informal participation (once a month or less, once a week or less, and almost every day or two to three times a week) produced 12 categorical variables. Individuals who worked < 35 h per week and have a high degree of informal participation had an odds ratio (OR) of WLB of 1.0. R software (version 4.2; R Foundation for Statistical Computing, Vienna, Austria) was used to perform the statistical analyses.

## Results

### Comparison of sociodemographic characteristics, work- and life-related factors, and health-related factors by age

Participants were divided into 55–59, 60–69, 70–79, and > 80 years age groups. The demographic, work- and life-related factors, and health characteristics of participants by age are shown in Table 1. There were 727 (12.6%), 1,986 (34.5%), 1,922 (33.4%), and 1,116 (19.4%) participants aged 55–59, 60–69, 70–79, and ≥ 80 years, respectively. Significant differences in sociodemographic characteristics, work- and life-related factors, and health status were seen among age groups (all *p* < .05). Compared to younger participants, those in their 60 and 70 s were more likely to be widowed, separated, or divorced, had fewer years of education, had a lower income, and had fewer assets. Participants in the youngest age group were the most likely to be employed and have long working hours. Participants in the oldest age group were the least likely to participate in social activities but had the most contact with friends and relatives.

**Table 1:** Comparison of sociodemographic characteristics and work-, life-, and health-related factors among age groups, n (%)

Variables	ALL ^a^ n = 5,751	55–59 years 727 (12.6)	60–69 years 1,986 (34.5)	70–79 years 1,922 (33.4)	≥ 80 years 1,116 (19.4)	*p* ^*b*^
**Socioeconomic characteristics**						
**Sex**						
Male	2,492 (43.3)	298 (41.0)	905 (45.6)	841 (43.8)	448 (40.1)	0.02
Female	3,259 (56.7)	429 (59.0)	1,081 (54.4)	1,081 (56.2)	668 (59.9)	
**Education level**						
Primary school	2,537 (44.1)	77 (10.6)	563 (28.3)	1,072 (55.8)	825 (73.9)	< 0.01
Middle school	1,108 (17.7)	127 (17.5)	474 (23.9)	310 (16.1)	107 (9.6)	
High school	1,689 (29.4)	410 (56.4)	748 (37.7)	400 (20.8)	131 (11.7)	
College or higher	570 (8.8)	113 (15.5)	201 (10.1)	140 (7.3)	53 (4.7)	
**Marital status**						
Separated, divorced, or widowed	1,475 (25.6)	78 (10.7)	289 (14.6)	534 (27.8)	574 (51.4)	< 0.01
Married	4,276 (74.4)	649 (89.3)	1,697 (85.4)	1,388 (72.2)	542 (48.6)	
**Income (quartile)**						
Q1 (lowest)	1,406 (24.4)	210 (28.9)	528 (26.6)	393 (20.4)	275 (24.6)	< 0.01
Q2	1,483 (25.8)	61 (8.4)	323 (16.3)	626 (32.6)	473 (42.4)	
Q3	1,366 (23.8)	109 (15.0)	442 (22.3)	550 (28.6)	265 (23.7)	
Q4 (highest)	1,496 (26.0)	347 (47.7)	693 (34.9)	353 (18.4)	103 (9.2)	
**Assets (quartile)**						
Q1 (lowest)	1,418 (24.7)	141 (19.4)	395 (19.9)	469 (24.4)	413 (37.0)	< 0.01
Q2	1,463 (25.4)	178 (24.5)	475 (23.9)	543 (28.3)	267 (23.9)	
Q3	1,484 (25.8)	197 (27.1)	559 (28.1)	467 (24.3)	261 (23.4)	
Q4 (highest)	1,386 (24.1)	211 (29.0)	557 (28.0)	443 (23.0)	175 (15.7)	
**Region**						
Metropolis	2,390 (41.6)	341 (46.9)	881 (44.4)	744 (38.7)	424 (38.0)	< 0.01
Medium-sized city	1,829 (31.8)	253 (34.8)	665 (33.8)	596 (31.0)	315 (28.2)	
Small-sized city	1,532 (26.6)	133 (18.3)	440 (22.2)	582 (30.3)	377 (33.8)	
**Religious**						
No	3,299 (57.4)	458 (63.0)	1,111 (55.9)	1,067 (55.5)	663 (59.4)	< 0.01
Yes	2,452 (42.6)	269 (37.0)	875 (44.1)	855 (44.5)	453 (40.6)	
**Family ties**						
**Number of children who they meet weekly**						
None	4,561 (79.3)	638 (87.8)	1,592 (80.2)	1,467 (76.3)	864 (77.4)	< 0.01
≥ 1	1,190 (20.7)	89 (12.2)	394 (19.8)	455 (23.7)	252 (22.6)	
**Number of children who phone or mail weekly**						
None	1,853 (32.2)	353 (48.6)	648 (32.6)	526 (27.4)	326 (29.2)	< 0.01
≥ 1	3,898 (67.8)	374 (51.4)	1,338 (67.4)	1,396 (72.6)	790 (70.8)	
**Work-related factors**						
**Working hours (per week)**						
1–34	687 (11.9)	96 (13.2)	293 (14.8)	243 (12.6)	55 (4.9)	< 0.01
35–54	1,061 (18.4)	338 (46.5)	536 (27.0)	164 (8.5)	23 (2.5)	
≥ 55	378 (6.6)	80 (11.0)	203 (10.2)	80 (4.2)	15 (1.3)	
0 (not working)	3,625 (63.0)	213 (29.3)	954 (48.0)	1,435 (74.7)	1,023 (91.7)	
**Job-related demands**						
No	5,322 (92.5)	609 (83.8)	1,768 (89.0)	1,846 (96.0)	1,099 (98.5)	< 0.01
Yes	429 (7.5)	118 (16.2)	218 (11.0)	76 (4.0)	17 (1.5)	
**Physical requirements of the job**						
No	4,267 (74.2)	387 (53.2)	1,264 (63.6)	1,570 (81.7)	1,045 (93.7)	< 0.01
Yes	1,484 (25.8)	340 (46.8)	722 (36.4)	352 (18.3)	70 (6.3)	
**Caregiving**						
No	5,651 (98.3)	722 (99.3)	1,959 (98.6)	1,892 (98.4)	1,078 (96.6)	< 0.01
Yes	100 (1.7)	5 (0.7)	27 (1.4)	30 (1.6)	38 (3.4)	
**Social engagement**						
**Formal participation**						
High	1,184 (20.6)	218 (30.0)	508 (25.6)	354 (18.4)	104 (9.3)	< 0.01
Medium	3,191 (55.5)	420 (57.8)	1,181 (59.5)	1,046 (54.5)	544 (48.7)	
Low	1,376 (23.9)	89 (12.2)	297 (15.0)	522 (27.2)	468 (41.9)	
**Informal participation**						
High	2,426 (42.2)	257 (35.4)	779 (39.2)	861 (44.8)	529 (47.4)	< 0.01
Medium	1,761 (30.6)	270 (37.1)	643 (32.4)	559 (29.1)	289 (35.9)	
Low	1,564 (27.2)	200 (27.5)	564 (28.4)	502 (26.1)	298 (26.7)	
**Health-related factors**						
**Self-rated health**						
Poor or average	4,184 (72.8)	337 (46.4)	1,268 (63.8)	1,561 (81.2)	1,018 (91.2)	< 0.01
Good	1,567 (27.2)	390 (53.6)	718 (36.2)	361 (18.8)	98 (8.8)	
**Work limitations due to health problems**						
No	3,745 (65.1)	612 (84.2)	1,498 (75.4)	1,179(61.3)	456 (40.9)	< 0.01
Yes	2,006 (34.9)	115 (15.8)	488 (24.6)	743 (38.7)	660 (59.1)	
**Instrumental ADL limitations**						
No	5,237 (91.1)	702 (96.6)	1,878 (94.6)	1,767 (91.9)	890 (79.7)	< 0.01
Yes	514 (8.9)	25 (3.4)	108 (5.4)	155 (8.1)	226 (20.3)	
**Cognitive problems**						
Normal cognition	4,281 (74.4)	693 (95.3)	1,756 (88.4)	1,317 (68.5)	515 (46.1)	< 0.01
Possible dementia	1,470 (25.6)	34 (4.7)	230 (11.6)	605 (31.5)	601 (53.9)	
**Number of chronic diseases**						
None	1,726 (30.0)	442 (60.8)	741 (37.3)	373 (19.4)	170 (15.2)	< 0.01
1	1,772 (30.8)	207 (28.5)	679 (34.2)	604 (31.4)	282 (25.3)	
≥ 2	2,253 (39.2)	78 (10.7)	566 (28.5)	945 (49.2)	664 (59.5)	
**BMI (kg/m** ^**2**^ **)**						
< 23	2,703 (47.0)	338 (46.5)	857 (43.2)	889 (46.3)	619 (55.5)	< 0.01
23–24.9	1,496 (26.0)	207 (28.5)	564 (28.4)	499 (26.0)	226 (20.3)	
≥ 25	1,552 (27.0)	182 (25.0)	565 (28.4)	534 (27.8)	271 (24.3)	
**Depressive symptoms**						
No	3,757 (65.3)	558 (76.8)	1,405 (70.7)	1,208 (62.9)	586 (52.5)	< 0.01
Yes	1,994 (34.7)	169 (23.2)	581 (29.3)	714 (37.1)	530 (47.5)	
**Smoking**						
Non or former smoker	5,134 (89.3)	617 (84.9)	1,705 (85.9)	1,757 (91.4)	1,055(94.5)	< 0.01
Current smoker	617 (10.7)	110 (15.1)	281 (14.1)	165 (8.6)	61 (5.5)	
**Alcohol consumption**						
No	2,893 (50.3)	290 (39.9)	899 (45.3)	1,030 (53.6)	674 (60.4)	< 0.01
Yes	2,858 (49.7)	437 (60.1)	1,087 (54.7)	892 (46.4)	442 (39.6)	
**Exercise**						
No	3,706 (64.4)	451 (62.0)	1,217 (61.3)	1,203 (62.6)	835 (74.8)	< 0.01
Yes	2,045 (35.6)	276 (38.0)	769 (38.7)	719 (37.4)	281 (25.2)	

*Note*: ^a^ Percentages are the column percentages of each variable. ^b^ Chi-squared test

*Significance levels*: ^*^p < .05, ^**^p < .01

### Associations among working hours, social engagement, and depressive symptoms

Compared to participants working for 1–34 h per week, those working for 35–54, ≥ 55, or 0 h per week had ORs of depressive symptoms of 1.34 (95% confidence interval [CI] = 1.03–1.71), 1.49 (95% CI = 1.09–2.04), and 1.74 (95% CI = 1.32–2.30), respectively, even after adjusting for covariates (Table 2). Informal participation was also associated with depressive symptoms (OR = 1.57, 95% CI = 1.35–1.83 for low level of contact). There was no sex difference in the effects of working hours (*p* = .08) and informal participation (*p* = .70) on depressive symptoms. Formal participation was not significantly associated with depressive symptoms (CI = 0.97–1.36 and 95% CI = 0.96–1.47 for one and none, respectively).

**Table 2:** Associations of the number of working hours and degree of social engagement with depressive symptoms

Variables	Odds ratio (95% confidence interval)
**Marital status**	
Separated, divorced, or widowed	1.00
Married	0.57 (0.49–0.66)^**^
**Income (quartile)**	
Q1 (lowest)	1.00
Q2	0.86 (0.73–1.02)
Q3	0.82 (0.69–0.97)^*^
Q4 (highest)	0.55 (0.45–0.67)^**^
**Assets (quartile)**	
Q1 (lowest)	1.00
Q2	0.77 (0.65–0.91)^**^
Q3	0.75 (0.63–0.89)^**^
Q4 (highest)	0.82 (0.68–0.99)^*^
**Residential area**	
Metropolis	1.00
Medium-sized city	1.26 (1.10–1.45)^**^
Small-sized city	0.77 (0.66–0.91)^**^
**Work-, life-, and health-related factors**	
**Working hours (per week)**	
1–34	1.00
35–54	1.34 (1.03–1.71)^*^
≥ 55	1.49 (1.09–2.04)^*^
0 (not working)	1.74 (1.32–2.30)^**^
**Job-related demands**	
No	1.00
Yes	1.49 (1.16–1.92)^**^
**Physical requirements of the job**	
No	1.00
Yes	1.11 (0.86–1.39)
**Caregiving**	
No	1.00
Yes	2.65 (1.71–4.10)^**^
**Formal participation**	
High (involvement in two or more activities)	1.00
Medium (involvement in one activity)	1.15 (0.97–1.36)
Low (no involvement)	1.19 (0.96–1.47)
**Informal participation**	
High (almost every day or 2–3 times a week)	1.00
Medium (once a week or twice a month)	1.00 (0.86–1.16)
Low (once a month or less)	1.57 (1.35–1.83)^**^
**Self-rated health**	
Poor or average	1.00
Good	0.65 (0.56–0.76)^**^
**Work–related limitations due to health problems**	
No	1.00
Yes	2.05 (1.80–2.34)^**^
**Limitations in instrumental ADL**	
No	1.00
Yes	1.28 (1.04–1.58)^**^
**Cognitive problems**	
Normal cognition	1.00
Possible dementia	1.69 (1.46–1.96)^**^
**Number of chronic diseases**	
None	1.00
1	1.06 (0.90–1.25)
≥ 2	1.34 (1.13–1.57)^**^

*Notes*: Models were adjusted for age and all variables listed in Table 1

*Significance levels*: ^*^p < .05, ^**^p < .01

Marital status (OR = 0.57, 95% CI = 0.49–0.66), household income (OR = 0.82, 95% CI = 0.69–0.97 and OR = 0.55, 95% CI = 0.45–0.67 for Q3 and Q4, respectively), assets (OR = 0.77, 95% CI = 0.65–0.91; OR = 0.75, 95% CI = 0.63–0.89; and OR = 0.82, 95% CI = 0.68–0.99 for Q2–4, respectively), residential area (OR = 1.26, 95% CI = 1.10–1.45 and OR = 0.77, 95% CI = 0.66–0.91 for medium- and small-sized cities, respectively), job-related demands (OR = 1.49, 95% CI = 1.16–1.92), and caregiving (OR = 2.65, 95% CI = 1.71–4.10) were associated with depressive symptoms. Finally, SRH (OR = 0.65, 95% CI = 0.56–0.77), work-related limitations due to health problems (OR = 2.05, 95% CI = 1.80–2.34), limitations in instrumental ADL (OR = 1.28, 95% CI = 1.04–1.58), cognitive problems (OR = 1.69, 95% CI = 1.46–1.96), and number of chronic diseases (OR = 1.34, 95% CI = 1.13–1.57 for ≥ 2 diseases) were related to depressive symptoms.

### Association between depressive symptoms and a composite index based on the number of working hours and the extent of social engagement

The composite index, which is based on the number of working hours and degree of social engagement, was found to be related to depressive symptoms, as shown in Table 3. Depressive symptoms were lowest among those who worked < 35 h and had a high level of informal participation, whereas non-working individuals with lack of informal participation had the highest rate of depressive symptoms (OR = 2.72, 95% CI = 1.89–3.91). Moreover, among working individuals, those who worked longer hours tended to experience more depressive symptoms compared to those who worked < 35 h. However, this positive relationship between working hours and depressive symptoms was weak and statistically insignificant for individuals with a high level of informal participation. For instance, the OR was only modest at 1.12 and not significant (CI = 0.76–1.65) for individuals who worked 35–54 h per week and had a high level of informal participation. The corresponding OR estimate for individuals who worked 35–54 h per week and had a low level of informal participation was about twice as large at 1.99 and highly significant (CI = 1.33–2.97).

**Table 3:** Association of an index based on the number of working hours and degree of informal participation with depressive symptoms

	Working hours (per week)			
	1–34	35–54	≥ 55	0
**Informal participation**				
High	1	1.12	1.25	1.83
(almost every day or 2–3 times a week)		(0.76–1.65)	(0.78–2.01)	(1.29–2.61)^**^
Medium	0.92	1.64	1.95	1.65
(once a week or twice a month)	(0.52–1.32)	(1.12–2.41)^*^	(1.15–3.31)^**^	(1.15–2.37)^**^
Low	1.85	1.99	2.29	2.72
(once a month or less)	(1.17–2.93)^**^	(1.33–2.97)^**^	(1.34–3.92)^**^	(1.89–3.91)^**^

*Notes*: Models were adjusted for age and all variables listed in Table 1

*Significance levels*: ^*^p < .05, ^**^p < .01

## Discussion

We used nationally representative cross-sectional data from KLoSA to examine the associations among number of working hours, degree of social engagement, and depressive symptoms. Optimal number of working hours and degree of social engagement minimized the risk of depressive symptoms. In particular, older adults who worked for < 35 h per week were less likely to experience depressive symptoms compared to those who either did not work or worked for ≥ 35 h per week. Individuals with a high degree of informal participation (i.e., almost every day, or two to three times a week) were less likely to have symptoms compared to those with a low degree of informal participation (once a month or less). Also, an approach to WLB, based on the number of working hours and the degree of informal participation of older adults, was associated with a lower risk of depressive symptoms. Finally, depressive symptoms were least prevalent among those who worked for < 35 h per week and had a high degree of social engagement, whereas non-working individuals and those without sufficient social engagement showed the highest rates of depressive symptoms.

Our main contributions can be summarized as follows. First, most previous studies classified older adults as employed or unemployed, and as lacking or having social engagement, to evaluate the health effects of these factors, mainly because of a lack of data related to the working hours and degree of social engagement of older adults. There have been few studies of the effects of working hours and the extent of social engagement on health. Our data included the number of working hours (including zero-hour work) of older adults; therefore, we could examine the effects of working hours (in addition to social engagement) on depressive symptoms. Our data also allowed determination of the optimal number of working hours and degree of social engagement to minimize the risk of depressive symptoms. Second, most prior studies of WLB focused on the working-age population (aged < 65 years). Therefore, the concept of WLB for older adults is not well-established, as they are typically presumed to be retired. In this context, using both working hours and the degree of social engagement, we introduce a WLB concept for the older adult.

We presented the main analyses in two ways. First, we performed logistic regression analyses using the number of working hours and degree of social engagement as risk factors. Next, we created a composite variable for the WLB of older adults by combining the number of working hours with the degree of social engagement to represent aspects of WLB. We found that for older adults experiencing retirement, the optimal amount of working hours that contributed to the greatest protection from depressive symptoms was found in the first analysis. This finding is in contrast to those of studies conducted on working-age populations. Previous studies have shown that, among working-age Korean adults, depressive symptoms were more common in those who worked for < 40 h per week compared to those who worked for ≥ 40 h per week [60]. In Korea, workers who work for < 40 h per week are considered part-time workers and are provided with fewer benefits compared to those who work for 40 h; part-time workers also receive limited bonuses, experience job instability, and lack social support [61].

In the present study, older adults who worked for ≥ 35 h per week were more likely to experience depressive symptoms compared to those who worked for < 35 h per week. This is an unexpected finding, considering that the older adults who worked for < 35 h per week were employed in non-standard employment, similar to working-age individuals. Most previous studies of the effects of work on health outcomes of older adults did not distinguish between part- and full-time workers. However, a few studies have conducted subgroup analyses that showed that part-time workers had better subjective well-being than full-time workers [62]. This may be because full-time workers might be completely dependent on employment, while part-time workers may work to obtain the fringe benefits of jobs [62].

There are two possible reasons for the continued employment of older adults. First, they may be economically dependent on their salary. Second, older adults may continue to work for the purpose of self-actualization [63]. Monetary compensation is often considered the primary reason why older adults to continue work after retirement [64]. However, no study has specifically examined the proportion of older adults who are motivated to continue working for this reason. Several studies have suggested that factors other than monetary compensation can serve as motivators; for example, self-actualization and maintaining social engagement are significant drivers of post-retirement work [63, 64]. The relative importance of these factors may differ between individuals with low and high socioeconomic status [65]. While workers with a higher income and good pension may continue working for the purposes of self-actualization and maintaining social engagement, those with low socioeconomic status may continue to work due to financial difficulties, even if the work is physically demanding and unsatisfying [66]. Haider and Loughran (2001) revealed that older adults in the work force tended to be highly educated, wealthy, and healthy, and yet they earned a very low wage [67]. This is consistent with our finding that older adults who worked for < 35 h per week were less likely to experience depressive symptoms after adjusting for household income. Based on our findings, although older members of countries with low rates of poverty among older adults may have shorter optimal working hours, we believe that the act of working tends to have more positive effects than not working at all.

We also explored the relationship between degree of social engagement and depressive symptoms. A high level of informal participation was related to fewer depressive symptoms. However, there was no association between formal participation and depressive symptoms. An increasing number of studies suggest that social engagement is essential for the physical and mental health of older adults [68]. Previous studies have suggested that social connections promote psychological well-being by cultivating a sense of purpose and belonging, as well as providing emotional support at times of psychological distress [69]. However, most previous studies did not determine the optimal degree of social engagement. We found that individuals who engaged in informal participation more than twice a week (i.e., those with a sufficient degree of social engagement) were less likely to have depressive symptoms.

However, in this study, unlike informal participation, formal participation exhibited no association with depressive symptoms. In earlier studies that distinguished formal participation (such as participating in activities) from informal participation (engaging with friends, relatives, and neighbors), informal participation was more strongly associated with health-related variables than was formal participation [12, 70]. For example, the classic study by Lemon et al. (1972) demonstrated that informal social activity with friends positively correlated with life satisfaction, but formal social and solitary activities did not [12]. Moreover, a study that compared the findings of 42 cross-sectional and longitudinal works revealed that, of formal, informal, and solitary activities, informal participation exhibited the most substantial independent effect on subjective well-being [70]. These studies highlight that informal interactions with friends and relatives are often more personal and intimate than interactions within formal institutions. The emotions of understanding, trust, care, sympathy, protection, and comfort are more enhanced by informal participation [71, 72]. In essence, given the more intimate and frequent nature of informal participation, greater rewards and more specific role support are possible; informal participation is particularly beneficial [12]. Our finding that formal participation was insignificant but informal participation was significant align with those of previous research.

We constructed a composite variable by combining the number of working hours and the degree of social engagement, specifically informal participation, to represent aspects of WLB. Various combinations of working hours (0, < 35, 35–54, and ≥ 55 h) and informal participation levels (once a month or less, once a week or less, and almost every day or two to three times a week) produced 12 categorical variables. Older adults who worked for < 35 h per week and had a high degree of informal participation also had the best WLB, thus an OR for a good WLB of 1.0. Additionally, older adults who worked more hours were more likely to experience depressive symptoms compared to those with the optimal WLB. However, for individuals exhibiting high a level of informal participation, longer working hours did not significantly increase levels of depressive symptoms. This implies that for older adults who work many hours, it is important to maintain a reasonably high level of informal participation to manage the risk of depressive symptoms.

Several previous studies found that individuals tend to exhibit significant WLB improvements as they age [73, 74]. Theoretical and empirical researchers have suggested that as individuals progress through life, the demands they face diminish. According to Huffman (2013), the youngest and oldest age groups among all study participants exhibited the lowest levels of WFC [73]. In contrast, those between the ages of 25 and 45 years evidenced notably higher levels, perhaps because such individuals often work long hours and are at a time of life at which they are defined principally by their occupational identities. Moreover, subjects in this age group are commonly establishing families and raising young children.

However, Spieler (2018) moved beyond the demand perspective to explain the phenomenon using the concept of boundary management [74]. During the later stages of individual careers, the multiple demands imposed by both work and non-work coincide with noticeable declines in cognitive and physical resources [75]. To maintain efficacy in both the work and personal spheres despite these cognitive challenges, older workers tend to allocate their resources more selectively [76]. Although weak boundaries between the work and personal domains demand higher cognitive resources given the frequent transitions between these areas, strong boundaries reduce such transitions and mitigate potential conflicts between goals, thereby conserving cognitive energy. Essentially, creation of robust boundaries is a useful adaptive approach for older workers; this constitutes a thoughtful utilization of their cognitive resources. In this context, our study sought to define an optimal balance between work and life for individuals who are adapting to aging, using variables related to working hours and social engagement.

The characteristics of WLB among older persons reflect the nature of the aging process that requires adaptation in both work and social life. This highlights the need for a refined theoretical framework that comprehensively addresses aspects of aging, including retirement. With this in mind, we used the Retirement Adjustment from a Resource-Based Dynamic Process (RBDP) perspective [77]. This theory views retirement adjustment as a process wherein the adjustment level varies by individual resources and changes in several assets. At the core, this theory emphasizes that retiree adjustments are deeply influenced by shifts in their physical, cognitive, motivational, financial, social, and emotional resources [78]. If these resources remain stable over time, thus via maintenance of past routines and activities, no adaptation may be required. However, significant decreases in such resources may trigger maladjustment. Conversely, if retirement provides an opportunity to re-allocate resources, such as the cognitive efforts once used in a demanding job, this could shift overall well-being in a positive direction. The RBDP perspective considers psychological resilience to be a fundamental determinant of successful adjustment [77]. Aging typically involves transitions to retirement, and, during this time, individuals often face a decline in physical abilities and changes in social status and their social networks [79]. Although these transitions are natural, they shock many. How individuals perceive and respond to these shocks is crucial in terms of healthy aging [80]. This invokes the concept of resilience.

Resilience is described as the ability to adapt effectively in the face of adversity, trauma, tragedy, threats, or significant stressors [81]. Thus, resilience pertains to the response to shocks. The impacts of aging are not short-term events, rather including changes in social status on retirement, reduced social networks, and decline in physical function [79]. Hence, in the context of aging, resilience can be understood as the individual capacity to address changes in physical and mental abilities and social networks, thus the ability to form new relationships and adapt [82]. In other words, WLB in old age transcends the allocation of time between work and life that occupies younger subjects. WLB in older age encompasses the seeking of a new balance, or an adaptive change, to address the transitions of aging effectively. Work and life must be rebalanced in a manner that maximizes fulfilment rather than minimizes conflict. The fulfilment of multi-dimensional human needs is the basis of well-being, and requires WLB or “work-life harmony” [83]. The ability to make such adjustments is an essential element of resilience in old age.

By integrating the perspectives of work-life enrichment and RBDP during retirement, we found that the WLB of older people is better viewed as work-life fulfilment balance. This concept is applicable to all stages of life; it is an extension of enrichment theory. Previous studies focused on the working-age population and emphasized the importance of reducing excessive work demand for maintaining a good WLB. By contrast, in our study of the older population, very little or a complete lack of demands had a negative impact on depressive symptoms. In other words, for the working-age population, excessive work- and life-related demands are the primary causes of WLB, whereas the opposite is true for the older population, in whom a *lack* of job-related demands and social engagement are the primary causes of a WLB that can trigger health problems. Although for older adults, key life events, such as retirement and the death of friends significantly affect social engagement, and changes in later life such as poor health may restrict physical activities, it is important to maintain both work and social activities. We propose that the other side of the WLB (i.e., having minimum hours of work) could be at least equally important, to represent fulfillment of needs in work and life. The idea of WLB mainly applied to the working-age population can be developed for the aging population as work–life “fulfillment” balance, with a necessary modification due to a different underlying cause of the imbalance.

### Limitations

Our study had several limitations. First, the data were obtained through a cross-sectional survey; therefore, causality cannot be determined. However, previous longitudinal studies confirmed the direction of the association between working hours (and social engagement) with depressive symptoms [84, 85]. Second, the exposure and outcome variables were assessed based on self-reported data, which could have been influenced by the respondents’ personality or mood. Third, because we performed secondary analyses of existing data, the data could not be used to quantify WLB. We constructed a composite variable representing WLB by integrating the number of working hours and the degree of social engagement, specifically informal participation; these are aspects of WLB; however, it may be more accurately measured using well-established and validated multiple-item tools. Future studies should measure WLB in a more comprehensive manner based on additional factors, and in different age groups. Despite these limitations, our study contributes to the existing literature by creating a tool for measuring WLB in older adults. Our results may also guide future research on the WLB of older adults.

## Conclusions

Our findings imply that the risk of depressive symptoms in older adults may be minimized by optimizing both working hours and social engagement. We extend the existing concept of WLB that is suitable for older adults. We suggest that “fulfillment” for work and life and their balance are important for older adults and propose work–life “fulfillment” balance. Future studies should consider an extension (or modification) of the WLB for older adults and develop a direct and relevant measure thereof.

## Data Availability

The data used in this study are available at: http://survey.keis.or.kr.

## Abbreviations

WLB: Work-life balance
WFC: Work-family conflict
KLoSA: Korean Longitudinal Study of Aging
RBDP: Resource-Based Dynamic Process
